# Power Conservation through Energy Efficient Routing in Wireless Sensor Networks

**DOI:** 10.3390/s90907320

**Published:** 2009-09-11

**Authors:** Dionisis Kandris, Panagiotis Tsioumas, Anthony Tzes, George Nikolakopoulos, Dimitrios D. Vergados

**Affiliations:** 1 Dept. of Electronics, Technological Educational Institution of Athens, 12210 Athens, Greece; E-Mail: ee4194@teiath.gr (P.T.); 2 Dept. of Electrical and Computer Engineering, University of Patras, 26500 Rio, Greece; E-Mails: tzes@ece.upatras.gr (A.T.); gnikolak@ece.upatras.gr (G.N.); 3 Dept. of Informatics, University of Piraeus, 18534 Piraeus, Greece; E-Mail: vergados@unipi.gr

**Keywords:** wireless sensor networks, routing protocols, energy efficiency, hierarchical routing, scalability

## Abstract

The power awareness issue is the primary concern within the domain of Wireless Sensor Networks (WSNs). Most power dissipation ocurrs during communication, thus routing protocols in WSNs mainly aim at power conservation. Moreover, a routing protocol should be scalable, so that its effectiveness does not degrade as the network size increases. In response to these issues, this work describes the development of an efficient routing protocol, named SHPER (Scaling Hierarchical Power Efficient Routing).

## 1. Introduction

Recent technological advances have enabled the inexpensive development of sensor nodes which, despite their relatively small size, have extremely powerful sensing, processing and communication capabilities. The benefits arising from the collaborative use of such sensor nodes which are wirelessly interconnected, find an ever growing number of applications. A group of spatially distributed sensor nodes which are interconnected without the use of any wires constitutes a WSN [1,2].

The typical configuration of such a sensor node in a WSN includes single or multiple sensing elements, a data processor, communicating components and a power source of limited energy capacity. The sensing elements of such a sensor node perform measurements related to the conditions existing at its surrounding environment. These measurements are transformed into corresponding electric signals and are processed by the processing unit. A sensor node makes use of its communication components in order to transmit the data, over a wireless channel, to a designated sink point, referred to as a base station. The base station collects all the data transmitted to it in order to act as a supervisory control processor or an access point for a human interface or even as a gateway to other networks.

By using such a configuration as that described above and through the collaborative use of a large number of sensor nodes, a WSN is able to perform concurrent data acquisition of ambient conditions at various spots of interest located over wide areas. Nowadays, WSNs support an ever growing variety of applications including environment and habitat monitoring, surveillance and reconnaissance, home automation, biomedical applications, object tracking, traffic control, fire detection, inventory control, agriculture, machine failure diagnosis and energy management [1–7].

Despite however the profound advantages that the utilization of WSNs offers, it is strictly limited due to energy constraints. In fact, the most of energy expenditure of a sensor node takes place during wireless communication and the rest whilst sensing and data processing. Therefore, there is a need for eradication of energy inefficiencies at all layers of the protocol stack of sensor nodes [1]. More specifically, research on the network layer of the protocol stack focuses on the achievement of energy efficient route setup and consistent data communication from the sensor nodes to the base station in order to extend the network lifetime and thus prolong network connectivity [5]; this is why routing protocols in WSNs aim mainly at the attainment of power conservation.

However, the routing protocols developed for wired communication networks, which mainly aim at high Quality of Service (QoS), are considered to be inappropriate for WSNs for many reasons. Firstly, IP based schemes cannot `easily’ be applied in WSNs. Secondly, contrary to wired networks, most of the data flow in WSNs is directed from many sources to one single sink. Moreover, given that multiple sensor nodes located within the vicinity of a phenomenon may generate similar data, routing protocols in a WSN need to tackle the redundancy associated with the data traffic generated by similar measurements. Furthermore, sensor nodes are strictly constrained in terms of on board energy, storage and processing capacity.

For these reasons, many new protocols have been proposed for data routing in sensor networks. These protocols can be classified in several categories [8,9]. This article deals with ‘Hierarchical Routing’. The main idea in hierarchical schemes is that nodes are grouped into squads and perform data aggregation and multi hop communication. In this way, the number of transmitted messages to the base station is reduced for the benefit of system scalability and energy efficiency. On the other hand, the detrimental effect of all hierarchical routing protocols is the increase of the computational overhead and the corresponding delay.

Moreover, no matter which the routing protocol selected is, the power efficiency of the data routing process is affected by the route selection policy adopted at local level, i.e., the policy used in order to find the most appropriate path to route data from one node to another.

The rest of this work is organized as follows. In Section 2, a discussion on some of the most popular hierarchical routing protocols and route selection policies is offered. In Section 3, the system model adopted in the proposed SHPER protocol is described. In Section 4, the initialization and steady state phases of SHPER protocol are explained. The performance evaluation of SHPER takes place in Section 5 through the description of the simulation scenarios and the corresponding results. Finally, Section 6 concludes the article.

## 2. WSN Routing Protocols

This article aims at presenting in detail and thoroughly validating the enhanced work performed on the development of a hierarchical routing protocol for WSNs named SHPER (Scaling Hierarchical Power Efficient Routing), which was first introduced by Kandris *et al*. [10]. This section presents the most widely used protocols which have similar features with SHPER, when compared according to specific metrics such as classification, power usage, data aggregation ability, localization and scalability.

### 2.1. Hierarchical Routing Protocols

The LEACH protocol, which was introduced by Heizelman *et al*., is one of the most popular energy efficient hierarchical routing protocols proposed for sensor networks and has been utilized as the basis for many other subsequent protocols of such a kind [11]. In this protocol neighboring nodes dynamically form clusters in a distributed manner. One node per cluster is randomly chosen to be the cluster head. Each cluster head collects and aggregates all data it receives from its cluster nodes and forwards the aggregated data directly to the base station. The election of cluster heads is rotated among the cluster nodes and it is based on the determined *a priori* suggested proportion of cluster heads for the network and the number of times a node has been a cluster head so far. After the end of each round, the new elected cluster head sends to each one of the rest of its cluster nodes a consequent notification.

Given that in LEACH data collection is performed periodically, this protocol is supposed to be more suitable when there is a need for constant proactive monitoring by the WSN. However, in several cases, periodic data transmissions are needless, thus causing ineffectual expenditure of energy. Additionally, LEACH supposes that all nodes contain the same quantity of energy capacity at every election round. Furthermore, it is also presumed that every node has an adequate amount of transmission power in order to directly get in touch with the base station if required. Nevertheless, in most cases these hypotheses are unlikely.

A protocol which is considered to be an enhanced descendant of LEACH is PEGASIS, which was introduced by Lindsey and Raghavendra [12]. In this protocol the nodes are not grouped into clusters. Instead, chains of nodes are formed in a greedy way so that each node transmits and receives from a neighbor and eventually one node is selected to transmit to the base station. Although, PEGASIS achieves higher energy conservation when compared to LEACH, it suffers however from certain drawbacks. One of them is the excessive delay which is caused by distant nodes on the chain. Another weakness of the protocol has to do with the fact that the single leader can become a bottleneck for the network.

Hierarchical-PEGASIS, introduced by Lindsey *et al*, is an extension to PEGASIS [13]. It achieves a notable decrease of the delay incurred for packets during transmission to the base station by enabling simultaneous transmissions of data. However, both PEGASIS and Hierarchical-PEGASIS, like LEACH, are based on some unrealistic assumptions such as that all nodes have the same level of energy and are able to communicate with the BS directly.

TEEN is another widely referenced and extensively used energy efficient hierarchical routing protocol, proposed by Manjeshwar and Agrawal [14]. TEEN makes use of a hierarchical scheme along with a data centric mechanism. More precisely, in a way akin to that adopted by LEACH, adjacent nodes are dynamically grouped into clusters. Within every cluster, one of the cluster nodes is elected in turn to be the cluster head for a time interval called cluster period. At the beginning of every cluster period, each new elected cluster head broadcasts, to its cluster nodes, the values of two relative to the sensed attribute thresholds. The first of them is the so called hard threshold. It is the minimum value of the sensed attribute required to force a sensor node to activate its transmitter and transmit to the cluster head. The use of the hard threshold minimizes the number of transmissions for the reason that the nodes transmit only when the sensed attribute exceeds a critical value. The second one is the so called soft threshold. It represents a small alteration in the value of the sensed attribute that prompts the node to turn on its transmitter and transmit. The use of the soft threshold reduces further the number of transmissions, for the reason that in the presence of little or no change in the sensed attribute the sensor nodes do not transmit. Each cluster head collects and aggregates all data it receives from its cluster nodes and forwards the aggregated data either directly or indirectly to the base station.

TEEN has been designed so as to react to sudden alterations of the sensed attributes. Therefore, it is appropriate for reactive networks and time critical applications. However, TEEN is not suitable for applications which require periodic reports since the nodes may not transmit and thus the user may not get any data at all from the network if the thresholds are not reached.

APTEEN is an extension of TEEN, also proposed by Manjeshwar and Agrawal [15]. Although APTEEN makes use of a hierarchical scheme similar to that adopted by TEEN, it is however a hybrid protocol given that it enables both reactive and proactive ways of operation. More precisely, in APTEEN sensor nodes are required not only to respond instantly to time critical situations but to periodically send data too. The periodicity and the values of thresholds are adapted to the user needs and the type of the application.

APTEEN incorporates query handling in an efficient way. Three different query types are supported: historical, in order to explore past data, one-time, to take a snapshot view of the network and persistent, to observe an event for a period of time. Experimentally has been shown that APTEEN surpasses LEACH in terms of energy dissipation and network lifetime. However, the same simulation results proved that TEEN outperforms both protocols [15].

Many other hierarchical routing protocols have been proposed either in the past [8,9] or more recently [16,17]. Similarly, many alternative approaches have been proposed for energy conservation in WSNs, for instance through optimized clustering [18], or collaborative sensing and adaptive target estimation [19]. However, any reference to them is out of the scope of this article due to their dissimilarities from SHPER when compared according to specific features of classification.

### 2.2. Route Selection Schemes

Although a routing protocol may indicate the source and destination nodes associated with the routing procedures, it may not however specify the path through which a transmitting node should route its data to the receiving node. One of the main factors which influence the power awareness of a routing procedure is the selection of the most energy efficient route. In wired networks, where there are not any energy limitations, the hop count or/and the delay can be considered as suitable metrics for route selection. However, in WSNs the energy issue should be taken into account in order to find the most appropriate paths.

It is for this reason why many energy efficient route selection policies have been suggested. Singh *et al*. proposed the MBCR and MMBCR algorithms [20]. According to MBCR, the route having the maximum residual energy is selected. The main drawback of this scheme is that it can lead to decreased fairness among the WSN nodes since one node may be used extensively. The MMBCR algorithm suggests the selection of the path having the maximum residual energies of the nodes. The weakness of this algorithm is that it may lead to extremely lengthy paths.

Scott and Bambos proposed the MTPR algorithm, which recommends the selection of the route having the minimum total energy consumption during transmission [21]. The disadvantage of MTPR is that it cannot assure the life extension of each individual node because it does not take into consideration its residual energy.

Chang and Tassioulas proposed the MREPsum and MREPcap algorithms [22]. The first of them considers a cost function which represents the reciprocal of the residual energy of a node after the transmission of a packet, while the second uses as a cost function the energy expenditure per bit transmission divided by the residual energy.

Finally, Vergados *et al*. propose xMBCR and xMREPsum [23]. In these two schemes the cost functions proposed by MBCR and MREPsum correspondingly are raised to the power of a parameter, the value of which is adjusted in order to improve the performance of the two parent algorithms.

## 3. SHPER Protocol Model Description

This article proposes the enhanced integration of a hierarchical reactive routing protocol named SHPER, which aims at the achievement of energy conservation through the use of both an energy efficient routing strategy and a power aware route selection scheme.

**Figure 1.**
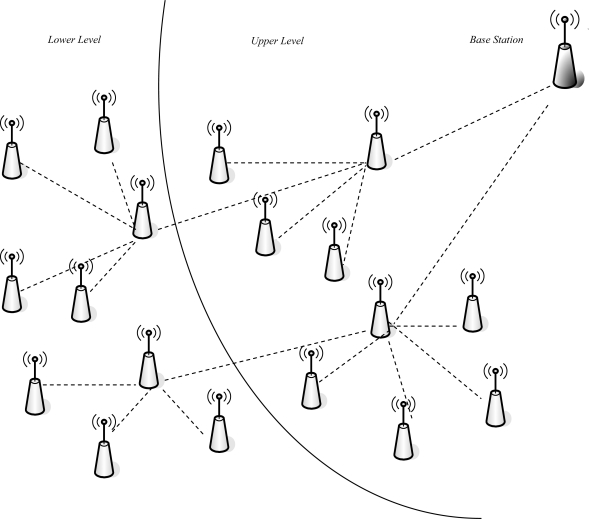
Graphical depiction of the model of the network field adopted in SHPER.

The model of the SHPER protocol supposes the coexistence of a base station and a set of homogeneous sensor nodes which are randomly distributed within a delimited area of interest. The base station is located at a distant position away from the sensor field. Both the base station and the set of the sensor nodes are supposed to be immobile (stationary). It is further assumed that the base station is able to transmit with high enough power to all the network nodes, due to its unlimited power supply. However, the network nodes are supposed to be energy constrained. All the network nodes are dynamically grouped into clusters. One of the nodes within every cluster is elected to be the cluster head. The cluster heads located close enough to the network base station are capable of direct transmission to the base station with reasonable energy expenditure. These cluster heads are supposed to be the *Upper Level* cluster heads. In a similar way, cluster heads located far away from the network base station, are considered to be the *Lower Level* cluster heads. This architecture scheme is illustrated in Figure 1. The adoption of such a scheme ensures the network scalability i.e., the ability of the routing procedures to sustain their performance unaffected by the increase of the overall network size. This is achieved because even nodes located at the most distant positions of the network field are able to route their messages to the base station by multihop routing via other neighboring nodes of the Lower Level and nodes of the Upper Level.

Moreover, contrary to other hierarchical protocols as those described in Section 2, the SHPER protocol specifies that the election of the cluster heads is not randomized. More precisely, the node elected to be the cluster head within each cluster is the one having the maximum residual energy. Furthermore, the route selection policy proposed takes into consideration both the residual energy of nodes and the energy consumption for all possible paths.

## 4. Protocol Operation Description

The operation of SHPER protocol, which is analytically explained in the rest of this section, consists of two phases, which namely are the initialization phase and the steady state phase.

At the very first round of the initialization phase, all nodes have to keep their receivers on (step 1). This is because the base station creates a TDMA (Time Division Multiple Access) schedule and requests the nodes to advertise themselves (step 2). The size of this schedule is equal to the number of the existing network nodes. In fact, this is the unique piece of information that the base station needs to have about the network field.

When the TDMA schedule is broadcasted, all nodes, during their allocated time slots, transmit their advertisement messages using the same transmission energy. In this way, each time a node advertises itself, the other nodes which receive this advertisement message estimate their comparative distance from this node, according to the power of the advertisement signal received (step 3).

As soon as the node advertisement procedure is accomplished, the base station chooses in a random way some of the nodes from which it has received an advertisement message to be the high level cluster heads. Similarly, the base station elects some of the nodes from which it has not received any advertisement message to be the low level cluster heads. The overall number of nodes which are assigned to be cluster heads is considered to be predefined (step 4).

After that, the base station broadcasts the identities (IDs) of the newly elected cluster heads. It also broadcasts the values of soft and hard thresholds (step 5). The use of these thresholds is explained in detail later on.

Next, each non cluster head node selects for the current round to participate into the cluster of the cluster head, whose advertisement message had been received before with the largest signal strength (step 6).

Since lower level cluster head nodes cannot transmit directly to the base station, it is necessary for them to route their messages via a path consisting of an upper level cluster head node and possibly other lower level cluster head nodes which are located closer to the network upper level. In this way however, there are various alternative paths that may me followed.

According to SHPER algorithm, each path *p* = (*c_1_*,*c_2_*,…,*c_n_*) between a lower level cluster head node *c_1_* and the base station *c_n_* that spans *n − 2* intermediate cluster head nodes *c_2_*,…,*c_n−1_*, is assigned with a corresponding value of the Routing Index *RI(p)*, which is shown in Equation (1):

(1) $$\mathrm{RI}(p)=\sum_{i=2}^{n-1}{\mathrm{Er}}_{i}-\sum_{i=1}^{n-1}E\left(c_{i},c_{i+1}\right)$$

where *Er_i_* denotes the residual energy of the cluster head node *c_i_*, and *E*(*c_i_*, *c_i+1_*) denotes the energy consumed for a message to be routed between two sequential cluster head nodes c*_i_* and *c_i+1_*.

If *A* is the set of all possible paths *p_l_* that can be followed for the transmission of the messages of a lower level cluster head to the base station, then the path *p_k_* selected is the one which satisfies the property given in Equation (2):

(2) $$p_{k}=\text{min}\{\mathrm{RI}\left(p_{l}\right):l\in A\}$$

By using Equations (1) and (2) each lower level cluster head defines the mostly energy efficient path to route its messages to the base station and selects the upper level cluster to belong to (step 7).

After each node has decided which cluster it belongs to, it informs its corresponding cluster head that is going to be a member of its cluster. Each node transmits this information back to its corresponding cluster head using a CSMA (Carrier-Sense Multiple Access) MAC protocol (step 8). That is why, cluster head nodes have to keep their receivers on during this specific step.

Each cluster head receives in this way all the messages from the nodes that want to be included in its cluster and creates, according to their quantity, a TDMA schedule of corresponding size (step 9).

Then each cluster head broadcasts this schedule back to the nodes in its cluster, in order to inform each node when it can transmit (step 10).

Next, the data transmission may start. Thus, during its allocated transmission time, each node sends to its cluster head quantitative data concerning the sensed events. In a way similar to that proposed in TEEN hard and soft thresholds are utilized in the SHPER protocol too. Thus, data are transmitted by a node to its cluster head within its corresponding time slot only when the presence of a sensed event is detected. This happens only when this node measures a value of the sensed attribute which is equal or greater than the hard threshold or when this value changes by an amount equal to or greater than the soft threshold. Along with the data concerning the sensed attributes the node transmits the current value of its residual energy. The radio of each non cluster head node can be turned off until the node’s allocated transmission time comes, thus minimizing energy dissipation in these nodes (step 11).

Each cluster head collects the data transmitted to it by the nodes of its own cluster (step 12). When all the data have been received, each cluster head performs signal processing functions to aggregate the data it has received along with its own data into a single composite message. This message contains the ID of the node having the highest residual energy among the cluster nodes, along with the most excessive (e.g., maximum) value of the sensed variable and the id of the node that has sensed it (step 13).

After each cluster head has created its aggregate message, it waits until its own time slot in order to transmit this aggregate message to the base station either directly, if this is possible, or via intermediate upper level cluster heads according to the path suggested by the use of Equations (1) and (2) (step 14).

Next, the base station collects all the messages transmitted to it (step 15).

After this step is completed, the initialization phase is ended and the steady state phase begins.

Hence, the base station determines the new cluster heads by using the data of the received messages. More specifically, the node having the highest residual energy in each cluster is elected to be the new cluster head. Moreover, the new values of soft and hard thresholds are defined (step16).

After that, all nodes switch their receivers on (step 17).

Finally, the base station broadcasts the ids of the new cluster heads along with the values of the thresholds (step 18).

Subsequently, each non cluster head node chooses the cluster it intends to belong to. The steady state phase is further carried on as a continuously recurrent execution of the procedures described above from step 6 to step 18.

**Figure 2.**
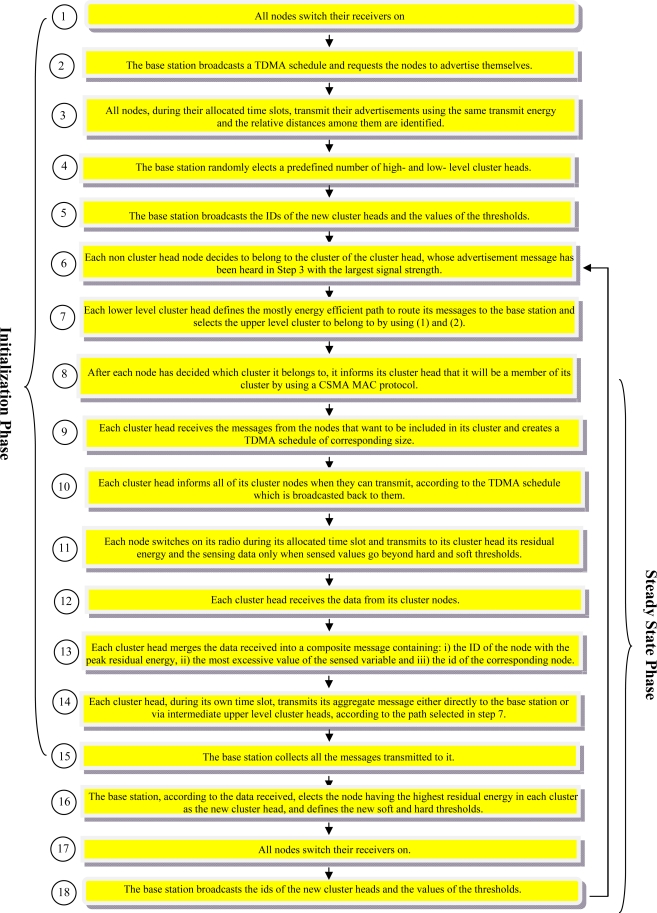
Synoptic overview of the algorithm chart of SHPER protocol.

A synoptic overview of the SHPER protocol algorithm is graphically depicted in Figure 2.

## 5. Protocol Performance Evaluation

Since, as it has been experimentally proven in [14] and [15], TEEN outperforms LEACH in terms of energy efficiency and network lifetime, SHPER was simulated against TEEN. The simulation was made by using the 1.5 version of TrueTime software, which is a Matlab/Simulink based simulator for real time control systems [24–26].

### 5.1. Simulation Setup Description

**Figure 3.**
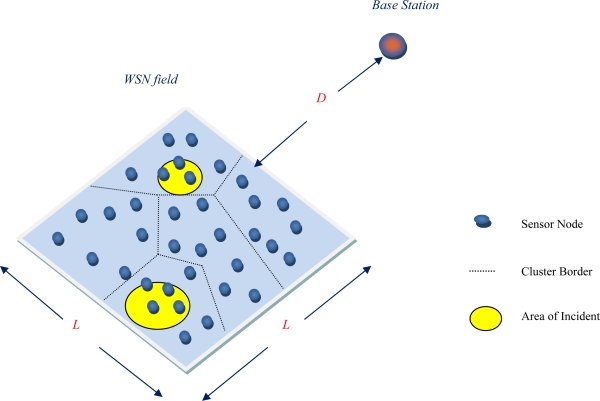
Graphical depiction of the architecture of the WSN scheme adopted.

The network used in the simulation scenario adopted is illustrated in Figure 3. It consists of 100 stationary sensors and a base station. More precisely, the nodes are supposed to be randomly deployed within the WSN field which is a square area of side *L* = 100 m. The base station is located at a distance *D* away from the closest point of this area. At each one of the simulation tests, *D* takes one value among 100, 200 or 300 m. The nodes transmit sensed data during their corresponding time slots, only when an incident is detected. This happens whenever the current value of the sensed attribute is greater than the hard threshold which is set to 100 and the current value of the sensed attribute differs from the sensed value by an amount equal to or greater than the soft threshold, which is set to 2. During simulation, incidents are generated at random locations within the network field, each of them concerning a region of arbitrary area.

The radio model that is used is the same adopted in [9]. By using this approach, an energy loss of *d^2^* due to channel transmission is assumed. The energy *E_Tx_(k,d)* that a node dissipates for the radio transmission of a message of *k* bits over a distance *d* is due to running both the transmitter circuitry [*E_Tx-elec_(k)*] and the transmitter amplifier [*E_Tx-amp_(k,d)*] and is given by:

(3) $$\begin{array}{l} E_{\mathrm{Tx}}\;(k,d)=E_{\mathrm{Tx–elec}}\;(k)+E_{\mathrm{Tx–amp}}\;(k,d)\Rightarrow \\ E_{\mathrm{Tx}}\;(k,d)=E_{\mathrm{elec}}\cdot k+\varepsilon_{\mathrm{amp}}\cdot k\cdot d^{2} \end{array}$$

where *E_elec_* is the transmitter circuitry dissipation per bit, which is supposed to be equal to the corresponding receiver circuitry dissipation per bit and *ε_amp_* is the transmit amplifier dissipation per bit per square meter. Similarly, the energy *E_Rx_(k)* dissipated by a node for the reception of a *k*-bit message is due to running the receiver circuitry [*E_Rx-elec_(k)*] and is given by:

(4) $$E_{\mathrm{Rx}}\;(k)=E_{\mathrm{Rx–elec}}\;(k)\Rightarrow E_{\mathrm{Rx}}\;(k)=E_{\mathrm{elec}}\cdot k$$

Moreover, a node dissipates additional sums of energy while being in idle state (equal to *E_elec_*) and while sensing (equal to 0.1·*E_elec_*). The initial node energy *Er_i_*(0) was set to 0.5 J for the half of the simulation tests performed. For the rest half of the simulation tests *Er_i_*(0) was supposed to take random values in the range between 0.1 J and 0.5 J.

It is assumed that each node has the ability of monitoring its residual energy. It is further assumed that the radio channel is symmetrical. Hence, the energy required to transmit a message from a source node to a destination node is equal to the energy required to transmit the same message from the destination node back to the source node for a given SNR.

Finally, it is assumed that the communication environment is both contention and error free. Thus there is no need for retransmission.

The parameters adopted during simulation tests along with their respective values are summarized in Table 1.

Finally, IEEE 802.11 b is the wireless communication standard used in the simulation tests performed. Generally, the selection of this standard enables the high rate transmission over long distances. Alternatively to this, IEEE 802.15.4 can be used in many applications. The great advantage of this standard is the achievement of low energy consumption and secure networking although the rates of transmission are not high.

Sensor node modules based on either of these standards can be used in order to perform experiments. However, the existence of hardware dependant particularities in such sensor modules influences the implementation of routing protocols. For instance, the antennas used in the sensor boards have a non perfect omnidirectional radiation pattern. The effect of this issue must be thoroughly investigated in order to bridge the discrepancies between the spherical radio model adopted in simulation tests and the physical reality of radio signals. An analytical research work on this topic has been performed by Zhou *et al*. [27].

### 5.2. Simulation Results Presentation

In total, 360 simulation tests were performed. In these tests the performance of SHPER protocol was investigated versus that of TEEN protocol, over different network topologies and various values of initial node energy and locations of the base station. More specifically, the number of nodes remaining alive over time was simulated for both protocols for the cases that the distance between the base station and the network field is either 100 m, or 200 m or 300 m.

**Figure 4.**
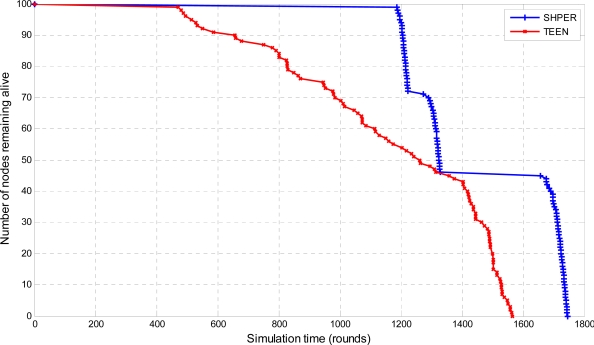
Comparative depiction of the nodes remaining alive over the simulation time steps in SHPER and TEEN [*Er_i_*(0) = 0.5 J, *D* = 100 m].

**Figure 5.**
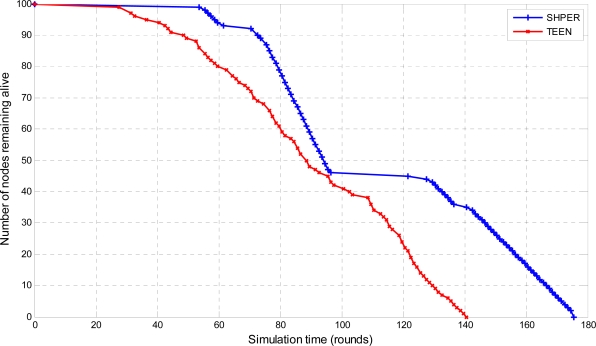
Comparative depiction of the nodes remaining alive over the simulation time steps in SHPER and TEEN, [*Er_i_*(0) = 0.5 J, *D* = 200 m].

**Figure 6.**
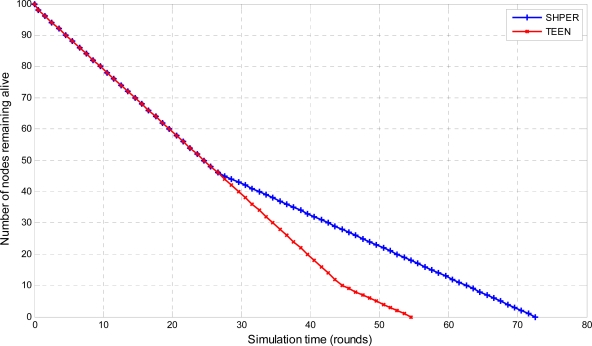
Comparative depiction of the nodes remaining alive over the simulation time steps in SHPER and TEEN [*Er_i_*(0) = 0.5 J, *D* = 300 m].

Moreover, the average percentages of the first node depletion time increase, the last node depletion time increase and the energy consumption decrease provided by using SHPER instead of TEEN, were calculated by using the Central Limit Theorem with 95% confidence intervals [28]. In the related figures, the upper, lower and central bars denote the upper bound, the lower bound and the center of the confidence intervals respectively. In the first half of the simulation tests, all of the nodes were considered to have their initial energy *Er_i_*(0) set to 0.5 J. The related simulation results concerning the number of nodes remaining alive over time are graphically displayed in Figures 4, 5 and 6.

**Figure 7.**
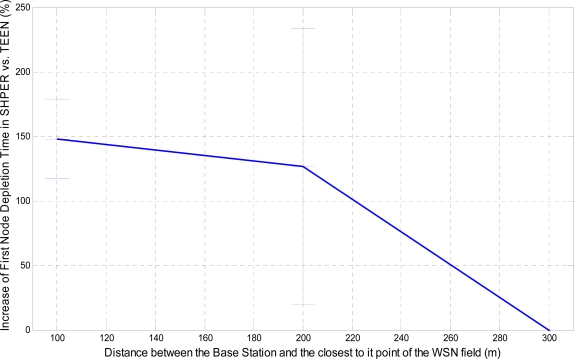
Percentage of the average increase of the time the first node depletes in SHPER opposed to TEEN over the distance of the base station from the WSN field, [*Er_i_*(0) = 0.5 J].

**Figure 8.**
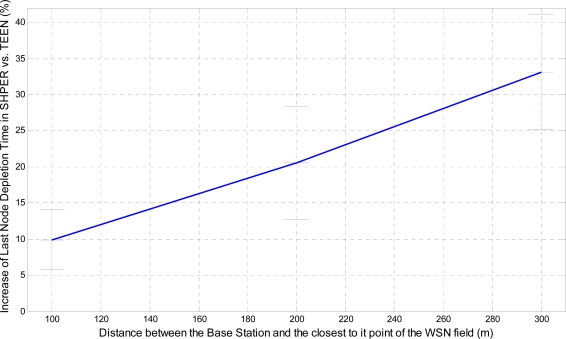
Percentage of average increase of the time the last node depletes in SHPER opposed to TEEN over the distance of the base station from the WSN field [*Er_i_*(0) = 0.5 J].

**Figure 9.**
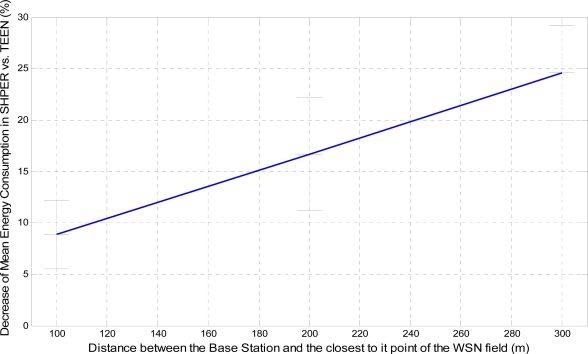
Percentage of average decrease of energy consumption in SHPER opposed to TEEN over the distance of the base station from the WSN field [*Er_i_*(0) = 0.5 J].

In a similar way, the corresponding graphical representation of the average percentages of the first node depletion time increase, the last node depletion time increase and the energy consumption decrease is performed in Figures 7, 8 and 9, respectively.

**Figure 10.**
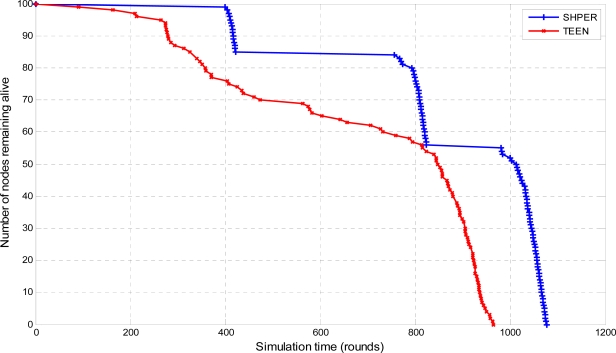
Comparative depiction of the nodes remaining alive over the simulation time steps in SHPER and TEEN [*Er_i_*(0)∈[0.1,0.5] J, *D* = 100 m].

**Figure 11.**
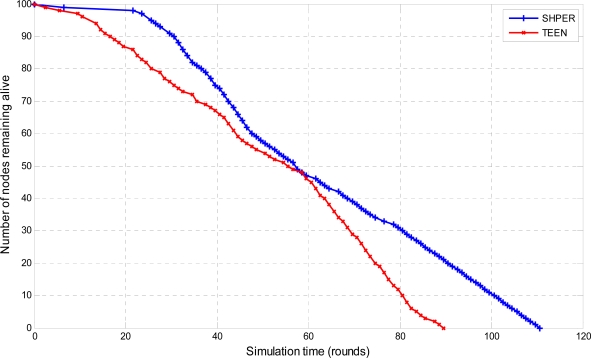
Comparative depiction of the nodes remaining alive over the simulation time steps in SHPER and TEEN [*Er_i_*(0)∈[0.1,0.5] J, *D* = 200 m].

**Figure 12.**
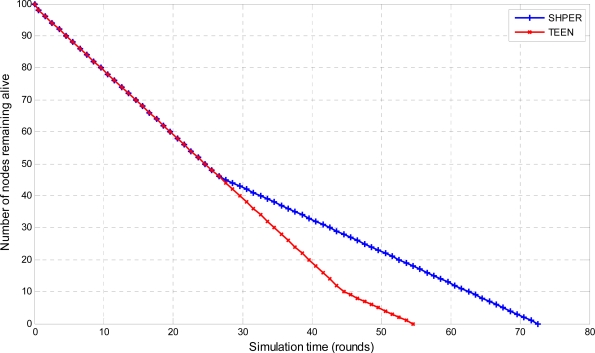
Comparative depiction of the nodes remaining alive over the simulation time steps in SHPER and TEEN [*Er_i_*(0)∈[0.1,0.5] J, *D* = 300 m].

In the last half of the simulation tests, the sensor nodes were considered to have their initial energy *Er_i_*(0) taking random values between 0.1 J and 0.5 J. The resultant simulation results concerning the number of nodes remaining alive over time are graphically displayed in Figures 10, 11 and 12.

**Figure 13.**
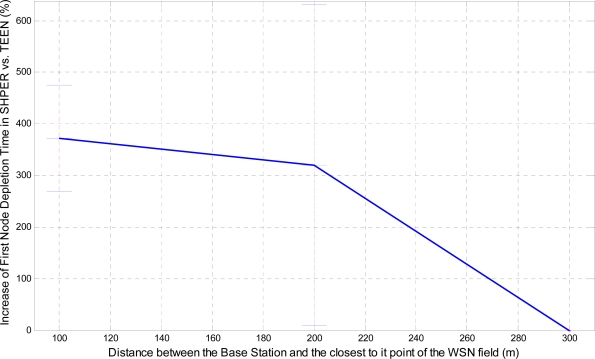
Percentage of the average increase of the time the first node depletes in SHPER opposed to TEEN over the distance of the base station from the WSN field (*Er_i_*(0)∈[0.1,0.5] J).

**Figure 14.**
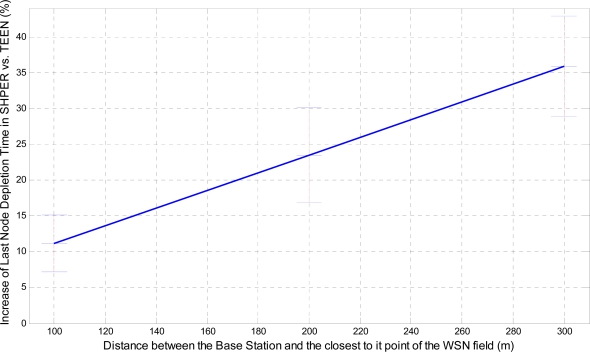
Percentage of average increase of the time the last node depletes in SHPER opposed to TEEN over the distance of the base station from the WSN field (*Er_i_*(0)∈[0.1,0.5] J).

**Figure 15.**
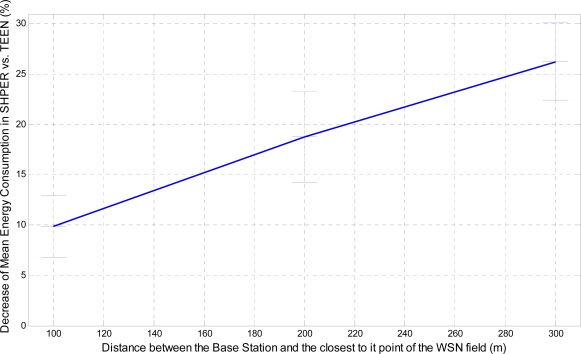
Percentage of average decrease of energy consumption in SHPER opposed to TEEN over the distance of the base station from the WSN field (*Er_i_*(0)∈[0.1,0.5] J).

Similarly, the related average percentages of the first node depletion time increase, the last node depletion time increase and the energy consumption decrease are depicted in Figures 13, 14 and 15.

### 5.3. Simulation Results Evaluation

The depletion of nodes over time is a typical measure of the energy efficiency of a routing protocol. Simulation tests show that SHPER protocol is particularly better than the TEEN protocol in retarding the time of node depletion, no matter what the distance between the base station and the nodes is.

More precisely, the total of the network nodes remain alive for a period which is considerably longer in SHPER than in TEEN when the base station is located relatively close to the WSN field. After this initial period, during which the energy load is shared among the whole of the nodes, the worn out nodes start to deplete until a new level of resistance is reached i.e., the routing is performed by other sets of nodes which belong to other levels of the network hierarchy. This is carried on until all nodes deplete. However, the overall network lifetime is prolonged when compared to TEEN. As distance between the base station and the network field increases, nodes get more rapidly exhausted but due to the more energy efficient routing in SHPER the power conservation becomes even more evident.

A more analytical description of the effects of the use of the two protocols in comparison may be derived by focusing on the first node depletion time, the last node depletion time and the average energy consumption. Table 2 summarizes arithmetically the simulation results which are illustrated in Figure 7, Figure 8 and Figure 9, concerning these three metrics in the case of uniform energy distribution.

In a similar way, Table 3 summarizes arithmetically the percentage enhancement caused by the use of SHPER instead of TEEN concerning the metrics of the first node depletion time, the last node depletion time and the average consumption in the case of non uniform energy distribution.

The examination of the data in Table 2 and Table 3 leads to the inference that the use of SHPER routing protocol instead of TEEN has the following consequences:

- The average time that the first node depletes is greatly increased when the base station is located relatively close to the WSN field. The extension of this time becomes gradually smaller as the distance between the base station and the WSN field increases. For long distances, there is not any extension of the first node depletion time, i.e., due to the remote location of the base station the first node which transmits to the base station becomes instantly exhausted. However, even in this case, the use of SHPER offers the accomplishment of more rational routing within the network thus leading to the retardation of the energy depletion.
- The average time that the last node depletes is exceptionally prolonged when the base station is located far away from the WSN field. The extension of this time becomes gradually smaller as the distance between the base station and the WSN field decreases. Actually, this is an absolutely normal after-effect of the corresponding great increase of the time the first node depletes in shorter distances. However, even in the case that the base station is located at its shortest distance from the network field, simulation results show that the extension of the overall network lifetime is still remarkable.
- The mean energy consumption is particularly decreased in all simulation scenarios examined, no matter what the position of the base station is. More precisely, the average reduction of the energy consumption is linearly increased with the increase of the distance between the base station and the WSN field. Hence, in the case where the base station is located far away from the WSN field the superiority of SHPER protocol over TEEN protocol becomes extraordinary. This result is really important since in real life applications it is both more common and desirable to have the base station located far away from the sensors. However, simulation results show that, even when the base station is located at its shortest distance from the network field, the reduction of the average energy consumption is still notable.

The comparison of Table 2 and Table 3 leads to the creation of Table 4. This table arithmetically demonstrates the percentage enhancement offered by using SHPER which concerns the metrics of the first node depletion time, the last node depletion time and the average consumption, in the case that nodes have different initial energy opposed to the case that all nodes have the same initial energy.

Many WSN hierarchical routing protocols are based on the assumption of an equal energy reserve for all nodes. However, in view of the fact that non uniform energy distribution is the normal case in real life applications, this basic assumption is apparently impractical. Table 4 makes it clear that the enhancement concerning all energy performance metrics which is provided by the SHPER protocol becomes even greater in the case of non uniform node energy distribution. Thus, the more unbalanced the energy distribution in a WSN is the most beneficial the use of SHPER becomes. Actually, this is a great advantage of SHPER since its algorithm is more realistic and effective. This enhances the belief that SHPER is indeed capable of being implemented in real test-bed environment in future work.

Therefore, SHPER achieves both remarkable power conservation and increased scalability through energy efficient multihop routing. Thus, it succeeds the main target that the majority of routing protocols proposed for WSNs aim at, which is the reduction of power consumption.

Actually, there are some schemes proposed that intent to address both energy efficiency and either security [29] or quality of service [30]. In the initial development of the SHPER protocol, no other network performance metric apart from energy efficiency has been taken into consideration. However, this is possible to be achieved. Kandris *et al*. have proposed a hybrid scheme for power and perceived QoS aware routing of multimedia content over Wireless Multimedia Sensor Networks [31]. This scheme comprises the advantages of both energy efficient routing and video packet scheduling resulting to increased efficiency in terms of power consumption along with received video distortion.

## 6. Conclusions

This work focused on hierarchical routing in WSNs through the use of a new enhanced reactive protocol, named SHPER, which aims at the achievement of power conservation through energy efficient routing.

Contrary to other popular routing protocols, SHPER performs the cluster leadership in a non randomized way, but by taking into account the residual energy of nodes. Thus, energy balance is achieved and the power depletion among the nodes is performed in a more even way.

Moreover, the data routing is based on a route selection policy which takes into consideration both the energy reserves of the nodes and the communication cost associated with the potential paths. A consequence of this is that optimal routes are selected and significant power conservation is achieved.

Furthermore, many other protocols are based on single hop routing. This kind of routing is impractical in networks deployed over wide regions. In contradiction to this, SHPER adopts both multihop routing and hierarchical architecture. In this way, the network scalability is retained.

An extended set of simulation tests were performed, comparing SHPER against TEEN, which is considered to be one of the most advanced hierarchical WSN protocols. The simulation comparison of these two protocols took place over several network configurations for various locations of the base station both with and without uniform distribution of node initial energy. Simulation tests compared the two protocols with reference to the number of nodes remaining alive over time, the first node depletion time, the last node depletion time and the average consumption.

Simulation results showed that SHPER outperforms TEEN concerning all of the energy performance metrics. Moreover, it was shown that the beneficial results offered by the use of SHPER are further increased when a non uniform initial node energy distribution is adopted. This makes SHPER even more preferable in real life applications where imbalance in energy distribution is the common case.

## Figures and Tables

**Table 1.** Simulation Parameters.

**Parameter**	**Value**
Network size	100 m × 100 m
Number of sensors	100
Distance from WSN field to the base station (*D*)	100 / 200/ 300 m
Node initial energy [*Er_i_*(0)]	0.5 J / 0.1–0.5 J
Transmitter circuitry dissipation (*E_elec_*)	50 nJ/bit
Amplifier dissipation (*ε_amp_*)	100 pJ/bit/m^2^
Data packet size	100 bits
Soft Threshold	2
Hard Threshold	100

**Table 2.** Percentage of change in WSN performance by the use of SHPER opposed to TEEN over the distance of the base station from the sensor field when the initial node energy is set to 0.5 J.

**Distance between the base station and the WSN field (m)**	**First node depletion time (%)**	**Last node depletion time (%)**	**Mean energy consumption (%)**
100	+148.09	+9.90	−8.89
200	+126.73	+20.53	−16.69
300	0	+33.09	−24.60

**Table 3.** Percentage of change in WSN performance by the use of SHPER opposed to TEEN over the distance of the base station from the sensor field when the initial node energy ranges from 0.1 to 0.5 J.

**Distance between the base station and the WSN field (m)**	**First node depletion time (%)**	**Last node depletion time (%)**	**Mean energy consumption (%)**
100	+372.50	+11.14	−9.88
200	+320.41	+23.46	−18.77
300	0	+35.91	−26.23

**Table 4.** Percentage of average enhancement in WSN performance by the use of SHPER in the case of a non uniform initial energy distribution opposed to the case of uniform initial energy distribution.

**Increase of first node depletion time (%)**	**Increase of last node depletion time (%)**	**Decrease of mean energy consumption (%)**
152.13	11.00	9.37
